# Phenotype and frequency of STUB1 mutations: next-generation screenings in Caucasian ataxia and spastic paraplegia cohorts

**DOI:** 10.1186/1750-1172-9-57

**Published:** 2014-04-17

**Authors:** Matthis Synofzik, Rebecca Schüle, Martin Schulze, Janina Gburek-Augustat, Roland Schweizer, Anja Schirmacher, Ingeborg Krägeloh-Mann, Michael Gonzalez, Peter Young, Stephan Züchner, Ludger Schöls, Peter Bauer

**Affiliations:** 1 Department of Neurodegenerative Diseases, Hertie-Institute for Clinical Brain Research, University of Tübingen, Hoppe-Seyler-Str. 3, 72076 Tübingen, Germany; 2 German Research Center for Neurodegenerative Diseases (DZNE), University of Tübingen, Tübingen, Germany; 3 Dr. John T. Macdonald Foundation Department of Human Genetics and John P. Hussman Institute for Human Genomics, University of Miami Miller School of Medicine, Miami, USA; 4 Institute of Medical Genetics and Applied Genomics, University of Tübingen, Tübingen, Germany; 5 Department of Neuropediatrics, University of Tübingen, Tübingen, Germany; 6 Department of General Pedatrics, Pediatric Endocrinology and Diabetology, University of Tübingen, Tübingen, Germany; 7 Department of Sleep Medicine and Neuromuscular Disorders, Neurology, University Hospital Münster, Münster, Germany

**Keywords:** Ataxia, Recessive ataxia, Spastic ataxia, Early onset ataxia, Hypogonadism, Genetics, Magnetic resonance imaging, Electrophysiology, Cognitive impairment, Hereditary spastic paraplegia

## Abstract

**Background:**

Mutations in the gene *STUB1,* encoding the protein CHIP (C-terminus of HSC70-interacting protein), have recently been suggested as a cause of recessive ataxia based on the findings in few Chinese families. Here we aimed to investigate the phenotypic and genotypic spectrum of *STUB1* mutations, and to assess their frequency in different Caucasian disease cohorts.

**Methods:**

300 subjects with degenerative ataxia (n = 167) or spastic paraplegia (n = 133) were screened for *STUB1* variants by whole-exome-sequencing (n = 204) or shotgun-fragment-library-sequencing (n = 96). To control for the specificity of *STUB1* variants, we screened an additional 1707 exomes from 891 index families with other neurological diseases.

**Results:**

We identified 3 ataxia patients (3/167 = 1.8%) with 4 novel missense mutations in *STUB1,* including 3 mutations in its tetratricopeptide-repeat domain. All patients showed evidence of pyramidal tract damage. Cognitive impairment was present only in one and hypogonadism in none of them. Ataxia did not start before age 48 years in one subject. No recessive *STUB1* variants were identified in families with other neurological diseases, demonstrating that *STUB1* variants are not simply rare polymorphisms ubiquitous in neurodegenerative disease.

**Conclusions:**

*STUB1*-disease occurs also in Caucasian ataxia populations (1.8%). Our results expand the genotypic spectrum of *STUB1*-disease, showing that pathogenic mutations affect also the tetratricopeptide-repeat domain, thus providing clinical evidence for the functional importance of this domain. Moreover, they further delineate the phenotypic core features of *STUB1*-ataxia. Pyramidal tract damage is a common accompanying feature and can include lower limb spasticity, thus adding *STUB1*-ataxia to the differential diagnosis of “spastic ataxias”. However, *STUB1* is rare in subjects with predominant spastic paraplegia (0/133). In contrast to previous reports, *STUB1*-ataxia can start even above age 40 years, and neither hypogonadism nor prominent cognitive impairment are obligatory features.

## Background

Whole exome-sequencing (WES) has led to the identification of many novel candidates genes for recessive ataxias. If confirmed in additional families and independent cohorts, these findings allow for a molecular diagnosis in the still large amount of patients with hitherto undefined recessive ataxia syndromes and for a delineation of phenotypic spectra in this highly heterogeneous group of diseases unified by common genetic causes [1,2]. For example, missense mutations in *STUB1* encoding the protein CHIP (C-terminus of HSC70-interacting protein), which functions as a molecular co-chaperone and ubiquitin E3 ligase and which interacts with several proteins involved in degenerative ataxia disease [3], have recently been suggested as a cause of early-onset recessive ataxia with hypogonadism [4,5]. This finding was supported by animal studies, but information about the core symptoms and the phenotypic and mutational spectrum is limited, as only very few families with *STUB1* mutations have been reported so far [4,5]. Moreover, the frequency in Caucasian disease populations is unknown, as *STUB1* has been reported only from Chinese subjects.

Using two large-scale screening approaches we here identified 3 novel *STUB1* families. These findings demonstrate that *STUB1*-ataxia occurs also in Caucasian populations. Moreover, they help to delineate the phenotypic core features and to expand the genetic spectrum. We show that *STUB1* mutations are frequently associated with pyramidal tract damage, including lower limb spasticity. However, in contrast to the first report of *STUB1*-ataxia [4], neither hypogonadism nor prominent cognitive impairment are core symptoms of the disease. Finally, our findings show that pathogenic mutations affect also the tetratricopeptide-repeat domain of *STUB1*, thus complementing experimental findings about the substantial functional importance of this domain.

## Methods

### WES of ataxia and spastic paraplegia subjects

N = 71 subjects with early-onset degenerative ataxia (age of onset <30 years) compatible with autosomal recessive inheritance (no ataxia in the parental generation), negative for trinucelotide repeat expansions causing Friedreich’s ataxia (FRDA) and spinoncerebellar ataxia (SCA) type 1, 2, 3, 6, 7, and 17, underwent whole exome-sequencing. Given the fact that pyramidal tract damage to the upper and lower limb was observed in the first reported *STUB1* family [4], we additionally exome-sequenced n = 133 subjects with pure or complicated hereditary spastic paraplegia (HSP), each compatible with autosomal recessive disease. WES was perfomed using the SureSelect Human All Exon 50 Mb kit (Agilent, Santa Clara, CA, USA) for in-solution enrichment and the Hiseq2000 instrument (Illumina, San Diego, CA, USA) as described previously ([6]; for details, see Additional file 1).

*Shotgun fragment library sequencing in ataxia subjects.* N = 96 additional *subjects with* early-onset degenerative ataxia (age of onset <30 years) negative for SCA 1,2,3,6,7,17 and FRDA repeats were sequenced for *STUB1* variants by shotgun fragment library sequencing by a Nextera approach (Illumina, San Diego, CA, USA). Briefly, the complete locus of the *STUB1* gene (2,654 bp including 5 prime and 3 prime untranslated region) was amplified in a single 3 kb PCR fragment and subjected to transposon-based library generation using the Nextera-XT chemistry with molecular identifiers (MID) according the manufacturer’s protocol. MID barcoded libraries were pooled and short fragments were removed before a pool of 96 samples was sequenced using Illumina MiSeq V2 chemistry with 2× 150 bp paired-end reads (Illumina, San Diego, CA, USA). After quality filtering, mapping and annotation was done with standard tools and public databases (hg19, stampy, dbSNP134, EVS, annovar). For all samples, the complete *STUB1* locus was covered >100 reads.

*Exclusion of additional mutations in known ataxia genes by high-coverage targeted enrichment (“ataxia panel”)*. The ataxia syndrome in patients identified to carry two *STUB1* variants by shotgun sequencing might be alternatively explained by an additional mutation in another ataxia gene. To reduce this likelihood, the *STUB1* patient identified by shotgun sequencing (subject 91078) was additionally screened for mutations in >120 known ataxia genes by a high coverage (>94% mean coverage) HaloPlex gene panel kit (Agilent, Santa Clara, CA, USA; details in Additional file 2).

*STUB1* seed analysis in >1700 exomes of other neurological diseases. To assess the general genetic variability of the *STUB1* gene unrelated to ataxia or spastic paraplegia, we screened *STUB1* mutations in n = 1707 additional exomes from 891 families with a wide range of other (i.e. non-ataxia, non-HSP) neurological phenotypes including e.g. Charcot-Marie-Tooth disease, amyotrophic lateral sclerosis, Alzheimer’s disease and others using relaxed filter conditions (MAF EVS6500 < 3%, QUAL > 30, GQ > 30, < 10 additional families in the in-house database GEM.app with the same segregating variant) [7].

### Ethics approval

This study was carried out in compliance with the Helsinki Declaration, and approved by the Institutional Review Board of the University of Tübingen, reference number 598/20118O1.

## Results

### Genetic findings

**Figure 1:**
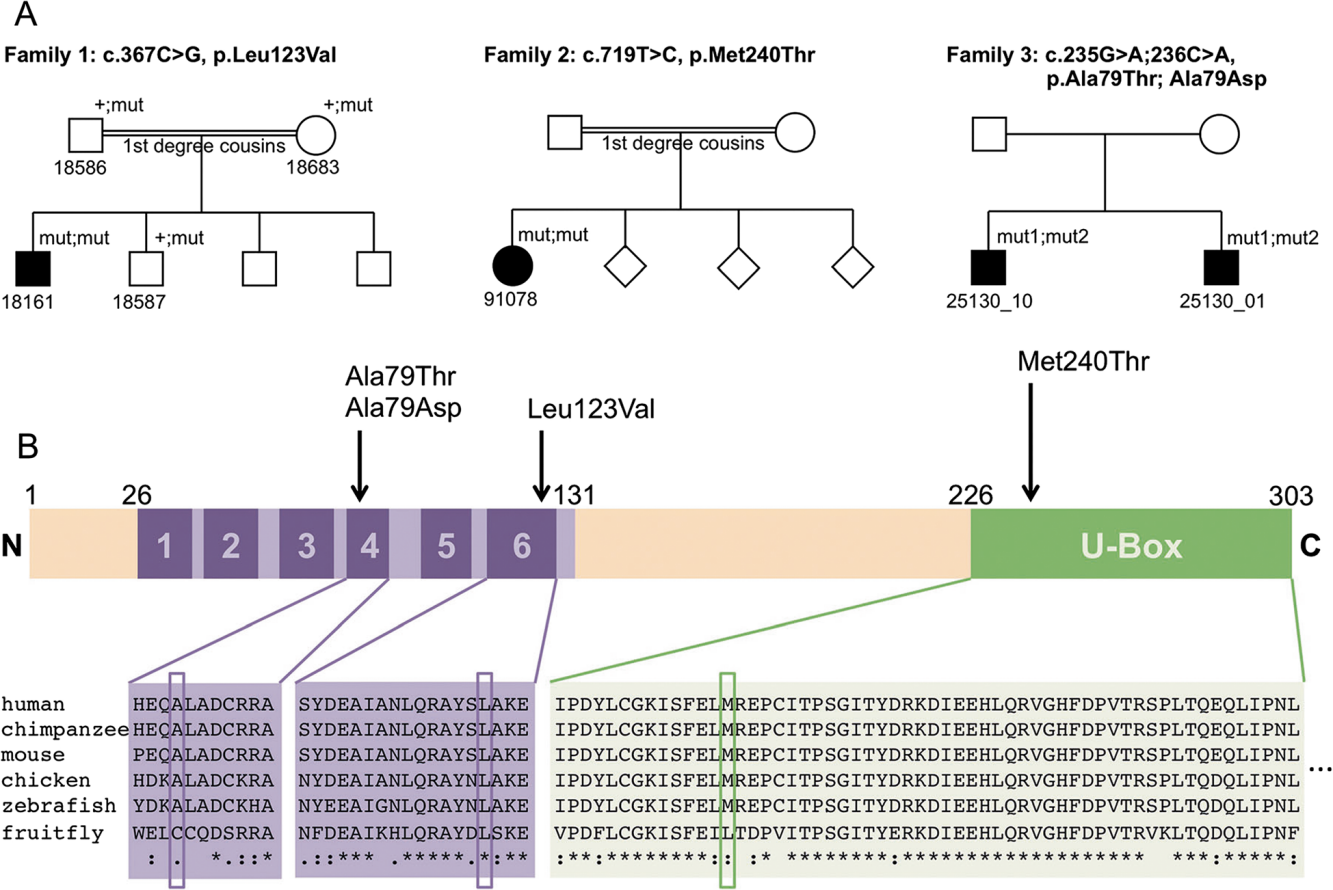
**Pedigrees of *STUB1 *index patients, and location and conservation of *STUB 1 *mutations. (A)** Pedigrees of families #1 and 2 reveal consanguinity with parents being first degree cousins. Family #3 shows two affected siblings. **(B)** The protein structure shows that the p.Met240Thr variant is located in the U-box domain of CHIP, whereas the p.Leu123Val, p.Ala79Thr and Ala79Asp variants are located in the tetratricopeptide-repeat (TPR) domain. All variants are highly conserved across species.

We identified 3 ataxia index patients with either homozygous mutations (c.367C > G, p.Leu123Val and c.719 T > C, p.Met240Thr in two patients with consanguineous parents) or compound heterozygous mutations (c.235G > A; c.236C > A, p.Ala79Thr; p.Ala79Asp in one patient with non- consanguineous parents) in the *STUB1* gene (transcript NM_005861). The 3 index families came from Germany, western Turkey, and Saudi Arabia, respectively. All 4 novel *STUB1* variants identified in these cases affect highly conserved amino acid residues, are predicted to be damaging by at least three *in silico* prediction algorithms, and were neither found in > 2,500 additional exomes of the Gem.app database (including 200 individuals of Turkish/Arab origin) [7] nor in the 6,500 exomes of the NHLBI Exome Variant Server (EVS) (NHLBI GO Exome Sequencing Project [ESP], Seattle, WA; URL: http://evs.gs.washington.edu/EVS/, accessed January 2014) (Table 1). All mutations were confirmed by Sanger sequencing (for electropherograms, see Additional file 3). The variants cosegregated with disease where affected siblings were available (family #3), and were present only in a heterozygous state in unaffected siblings (family #1) (Figure 1A). For two of the three families we were able to confirm localization of the variants in trans. In family #1 this was achieved by sequencing of the parents (Additional file 3), in family #3 manual inspection of the sequencing reads established compound heterozygosity (Additional file 4). No other significant variants in known ataxia genes were identified in any of the three index subjects by WES and HaloPlex panel, respectively (for a list of the remaining gene variants after filtering, see Additional file 5).

**Table 1:** ***STUB1 *** **mutations identified in this study**

**Subject ID**	**Phenotype**	**cDNA change**	**Protein change**	**GVS function**	**GERP**	**PolyPhen2**	**LRT**	**Mutation taster**	**NHLBI EVS MAF**	**GEM.app MAF**
**18161**	Ataxia with pyramidal damage	c.367C > G, c.367C > G	p.Leu123Val	Missense	3,6	1	D	D	0	0
**91078**	Ataxia with pyramidal damage	c.719 T > C, c.719 T > C	p.Met240Thr	Missense	4,3	0.99	D	D	0	0
**25130_01**	Spastic ataxia	c.235G > A,	p.Ala79Thr	Missense	4.3	0,996	D	D	0	0
		c.236C > A	p.Ala79Asp	Missense	4.3	0.996	D	D	0	0

*GVS*, Genome Variant Server; *GERP*, Genomic Evolutionary Rate Profiling; *LRT*, Likelihood Ratio Test; *D*, Damaging; *NHLBI EVS MAF*, Minor allele frequency in the NHLBI exome variant server; *Gem.app MAF*, Minor allele frequency in the in-house database Gem.app.

While the p.Met240Thr variant is located in the U-box domain of CHIP, close to the previously described Thr246Met variant [4], the p.Leu123Val, p.Ala79Thr and Ala79Asp variants are located in the tetratricopeptide-repeat (TPR) domain (Figure 1B).

The three *STUB1* index patients account for 3/167 = 1.8% of all ataxia patients in our cohort. No *STUB1* variants were found in HSP patients (0/133 = 0%). Screening of *STUB1* variants in 1707 additional whole exomes from families with other neurological phenotypes revealed 6 heterozygous *STUB1* variants (Additional file 6), but no other homozygous or compound heterozygous variants. This demonstrates that the recessive *STUB1* mutations observed in our target cohort are not simply rare polymorphisms ubiquitous in neurodegenerative disease.

### Phenotypic findings

**Figure 2:**
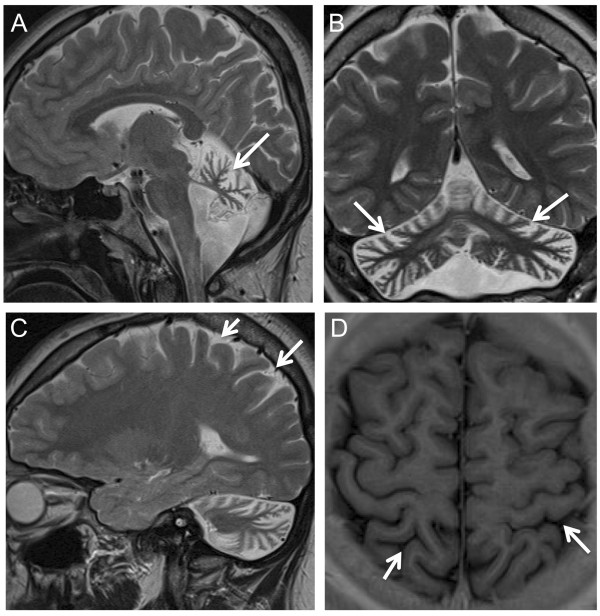
**Cerebral Magnetic Resonance Imaging in *STUB1 *ataxia.** T2 (**A**: sagittal; **B**, coronal; **C**, sagittal) and T1 weighted images (**D**, axial) of subject 18161 at age 17 years show marked atrophy of the cerebellar vermis (arrow, A) as well as the cerebellar hemispheres (arrows, **B**), and mild atrophy of the parietal cortex (arrows **C**, **D**).

All three index patients carrying two recessive *STUB1* mutations presented with cerebellar ataxia as the main and initial feature (for clinical details, see Table 2). Age of onset was highly variable, extending from age 2 years in subject 91078 to age 48 years in the brother of patient 25130_10. Evidence for pyramidal tract damage was found in all 4 patients. Two of them (25130_01 and his brother), showed clinical signs of pyramidal tract damage (increased lower limb tendon reflexes, non-exhaustible ankle clonus, mild lower limb spasticity) with normal central motor conduction times (CMCTs), while the other two subjects (91078 and 18161) displayed electrophysiological evidence of pyramidal tract damage with prolonged CMCTs. Subject 91078 showed myoclonic jerks in the face and the left arm. Distribution and phenomenology of the myoclonus point to a cortical origin, however no EMG-EEG recordings were available to confirm this hypothesis. Furthermore, subject 91078 had a mildly reduced vibration sense to the legs, which corresponded with electrophysiological evidence of sensory axonal peripheral neuropathy. No hypogonadism was clinically observed in any of the patients, and hormone investigations (available for 3/3 index patients) and testicular sonography findings (available for patient 18161) were normal. Whereas subject 91078 showed clinical signs of concentration difficulties, corresponding to mild deficits in working memory span (digit span and block span) and executive attention (Trail Making Test part B) on neuropsychological testing, no cognitive impairment was clinically observable in subjects 18161 and 25130_01. Neuropsychological testing by means of the German version of the Wechsler Intelligence Scale for Children [8] was normal in subject 18161. All patients showed marked cerebellar atrophy on MRI, subject 18161 presented also mild parietal cortical atrophy (Figure 2).

**Table 2:** **Clinical, MRI and lab findings of *STUB1 *** **mutation carriers**

**Subject**	**STUB1 mutation**	**Gender**	**Age at investigation [years]**	**Age at onset ataxia [years]**	**Tendon reflexes**	**Spasticity**	**Babinski reflex**	**Ankle clonus**	**Urge incontince**	**Hypogonadism**	**Cognitive impairment**	**SDFS**	**SARA**	**SPRS**	**Nerve conduction studies**		**Motor evoked potentials**	**MRI**	**Hormones**	**Testicular volume (sonography)**
															**Sensory**	**Motor**				
**18161**	**p.Leu123Val**	M	16	2	Normal	-	-	-	+	-	-	3	10	n.a.	Sural nerve: normal	Tibial nerve: normal	Prolonged CMCT to LE (18 ms)	Atrophy cerebellum and parietal cortex	Testosteron normal (236 ng/dl)	25 ml, each side
																			Estradiol normal (8,4 pg/ml	
																			LH normal (3,6 IU/l)	
																			FSH normal (0,7 IU/l)	
**91078**	**p.Met240Thr**	F	21	16	Normal	-	-	-	+	-	+	4	12	n.a.	Sural nerve: no SNAP	Tibial nerve: normal	Prolonged CMCT to UE (10 ms), not evoked to LE	Atrophy cerebellum	Estradiol normal (50 pg/ml)	n.a.
																			LH normal (8.3 mE/ml)	
																			FSH normal (3.7 mE/ml)	
**25130_01**	**p.Ala79Thr; Ala79Asp**	M	46	29	Increased	Lower limb, mild	-	+ bilateral	-	-	-	6	n.d.	38	n.d.	n.d.	Normal	Atrophy cerebellum	Testosteron (4.3 ng/ml)	n.d.
																			Estradiol normal (22 pg/ml9	
																			LH normal (9.6 mU/l)	
																			FSH normal (16.0 mU/ml)	
**25130_10**	**.p.Ala79Thr; Ala79Asp**	M	50	49	Increased	Lower limb. moderate	-	+ bilateral	+	-	-	3	14.5	5	n.d.	n.d.	Normal	Atrophy cerebellum	n.d.	n.d.

Legend: m, male; f, female; n.a., not applicable, n.d., not done; SARA, scale for the Assessment and Rating of Ataxia, reaching from 0 to 40, with higher scores indicating more severe ataxia [9]; scores < 3 points are considered unspecific. SPRS, spastic paraplegia rating scale, reaching from 0 to 52, with higher scores indicating more severe spastic paraplegia [10] (please note, however, that several items of the SPRS scale increase also with more severe ataxia); CMCT: Central motor conduction time. Reference values CMCT lower extremity (LE) < 16.0 ms; CMCT upper extremity (UE) < 9 ms. LE, lower extremity; UE, upper extremity; LH, luteinizing hormone; FSH, follicle-stimulating hormone; SNAP, sensory nerve action potential; MRI, magnetic resonance imaging. SDFS, Spinocerebellar degeneration functional score. This score was used to evaluate the disability stage from 1 to 7 (0: no functional handicap; 1: no functional handicap but signs at examination; 2: mild, able to run, walking unlimited; 3: moderate, unable to run, limited walking without help; 4: severe, walking with one stick; 5: walking with two sticks; 6: unable to walk, requiring wheelchair; 7: confined to the bed).

## Discussion

Recessive mutations in *STUB1* have recently been suggested as a cause of recessive ataxia [4,5], but knowledge about the phenotypic and genotypic spectrum is limited and no mutations have been reported in non-Chinese populations so far. Here we show that *STUB1*-ataxia is not limited to Chinese populations, but can be observed also in Caucasian ataxia patients (frequency: 1.8% of non-Friedreich recessive ataxias). Like in the previously reported Chinese *STUB1* patients [4,5], there was no obvious preference for female or male gender.

Our findings extend the genotypic and phenotypic spectrum of *STUB1*. The mutational spectrum is not limited to the U-box domain and its adjacent regions [4,5], but can include also the TPR domain, as we show for three mutations. This clinico-genetic finding complements recent experimental investigations demonstrating that not only the U-box, but also the TPR domain is crucial for adequate functioning of CHIP in the endoplasmatic reticulum protein quality control [11] and for mediating its interactions with chaperones [12].

Our findings show that - in contrast to the first report of *STUB1*-ataxia [4] (the second report provides no comment on the presence or absence of hypogonadism [5]) - hypogonadism is not an obligate core features of the disease. Likewise, also cognitive impairment is more infrequent than suggested by the initial report [4]. These findings are in line with other recently identified multisystemic recessive ataxias which extend along a continuous phenotypic spectrum and where hypogonadism or cognitive impairment are only variable and infrequent features (see e.g. *PNPLA6* ataxia [6]).

In contrast to hypogonadism and cognitive impairment, pyramidal tract damage seems to be a more common feature of *STUB1*-ataxia. It is present in all three of our families (clinically in patient 25130_01 and his brother; electrophysiologically in the other two index patients). The difference between clinical and electrophysiological findings of pyramidal tract damage in these patients does not necessarily indicate an inconsistency. Electrophysiological evidence of pyramidal damage in the absence of clinical pyramidal tract signs (as observed in 91078 and 18161) indicates *subclinical* pyramidal tract damage. Clinical pyramidal tract signs in the absence of electrophysiogical evidence of pyramidal damage (as observed in 25130_01 and his brother) is a feature commonly seen in hereditary spastic paraplegias [13], and most likely explained by predominantly axonal damage of the corticospinal tracts in these subjects [13]. Pyramidal tract signs were also observed in several of the previously reported *STUB1* families [4,5]. In sum, this frequent combination of ataxia with pyramidal tract damage indicates that *STUB1* ataxia should be added to the differential diagnosis of the rapidly increasing list of “spastic ataxia” spectrum disorders [14].

The clinical/electrophysiological observation of frequent pyramidal tract damage in *STUB1* also provides an important insight into the underlying pathophysiology: mutant CHIP seems to lead to a dysfunction not only of cerebellar neurons, but also of motor neurons. This clinical observation complements and corroborates findings from several molecular studies which indicate that CHIP acts as a common central hub in different pathways of a large variety of neurodegenerative diseases, including motor neuron diseases [15]. Acting as co-chaperone and ubiquitin ligase, CHIP is a central component of neuronal protein homeostasis [16]. It forms a multiheteromeric complex with other chaperons, which together selectively activate autophagic removal and degradation of misfolded proteins [17]. Apart from key ataxia proteins like ataxin-1 [12] or ataxin-3 [3,16], this degradation also includes proteins involved in motor neuron disease such as SOD1 proteins [17-19]. Thus, mutations in *STUB1*/CHIP, as observed in our patients, might lead to an impaired clearance of naturally occuring misfolded motor neuron proteins. This hypothesis stimulates future functional and pathology studies required to confirm this notion.

From clinical perspective it is interesting to note, however, that albeit upper motor neuron damage is common in *STUB1* disease, spastic paraparesis does not seem to be a predominant phenotype. This as indicated by the absence of *STUB1* mutations in our large HSP cohort.

## Conclusions

Our results show for the first time that *STUB1* ataxia occurs also outside of Chinese populations (frequency 1.8% of non-Friedreich recessive ataxias), and expand the mutational spectrum of this disease. They demonstrate a frequent association of cerebellar ataxia with upper motor neuron damage in *STUB1-*disease. At the same time, however, they show that *STUB1* is not observed in HSP patients. In contrast to the initial notion of *STUB1-*disease, hypogonadism seems to be an only infrequent feature of this disease.

## Abbreviations

ARCA: Autosomal recessive cerebellar ataxia; HSP: Hereditary spastic paraplegia; WES: Whole exome sequencing; TPR: Tetratricopeptide-repeat.

## Competing interests

The authors declare that they have no competing interests.

## Authors’ contributions

MS: design and conceptualization of the study, acquisition of data, analysis of the data, drafting the manuscript. RS: conceptualization of the study, acquisition of data, analysis of the data, revising the manuscript. MS, JG-A, RS, AS, IK-M, MG, PY, SZ, LS: acquisition of data, analysis and interpretation of the data, revising the manuscript. PB: design and conceptualization of the study, acquisition of data; analysis and interpretation of the data, revising the manuscript. All authors read and approved the final manuscript.

## Supplementary Material

Additional file 1 Whole exome sequencing methods. Click here for file

Additional file 2 Targeted enrichment of known ataxia genes (“ataxia panel”). Click here for file

Additional file 3 Electropherograms of Sanger Sequencing. Click here for file

Additional file 4 Sequencing Reads of an affected family member of family #3. Click here for file

Additional file 5 Genetic variants other than STUB1 identified in the STUB1 index patients by whole exome sequencing and the HaloPlex ataxia panel. Click here for file

Additional file 6 Heterozygous variants in STUB1 identified in a cohort of 1707 neurological disease controls (non-ataxia, non-HSP). Click here for file
