# Supplementary material for: Phenotype and frequency of STUB1 mutations: next-generation screenings in Caucasian ataxia and spastic paraplegia cohorts
Source: Orphanet J Rare Dis. 2014 Apr 17;9:57. doi: 10.1186/1750-1172-9-57 (PMC4021831; doi:10.1186/1750-1172-9-57)
Supplement: Additional file 1 — Whole exome sequencing methods. [file 1750-1172-9-57-S1.docx]

**Additional 1: Whole exome sequencing methods**

The SureSelect Human All Exon 50Mb kit (Agilent, Santa Clara, CA, USA) was used for in-solution enrichment and exome sequencing was performed using the Hiseq2000 instrument (Illumina, San Diego, CA, USA). Paired-end reads of 100 bp length were produced. BWA and GATK software packages [^1-3^](#_ENREF_1) were used to align sequence reads to the reference and call variant positions, respectively. All data were then annotated and imported into GEnomes Management Application (GEM.app), a web-based tool for next generation sequencing data analysis[^4^](#_ENREF_4) (genomics.med.miami.edu‎). An average of 73,609,687 sequence reads was produced per sample, 98.8% of which could be aligned to the targeted sequence. Mean coverage was 69-fold; 97% of the the coding sequence of STUB1 was covered >20 fold (see Figure below). Using the GEM.app analysis module ‘Genes Across Families’, we then filtered for non-synonymous homozygous or compound heterozygous variants in *STUB1*, with low frequency in public databases (minor allele frequency in dbSNP137 and NHLBI ESP6500 < 0.5%), moderate conservation (GERP score > 1 OR PhastCons score > 0.3) and moderate genotype quality (GATK quality index > 30 and genotype quality GQ > 30).


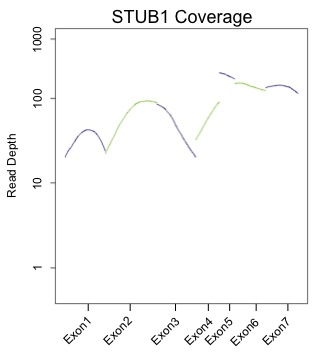


***Coverage of STUB1 by whole exome sequencing in a large-scale screening of various neurodegenerative phenotypes*.** The plot represents the average coverage across these samples. The alternating green and blue lines represent different exons in the gene. Please note the logarithmic scaling of the y-axis.
