# Supplementary material for: Phenotype and frequency of STUB1 mutations: next-generation screenings in Caucasian ataxia and spastic paraplegia cohorts
Source: Orphanet J Rare Dis. 2014 Apr 17;9:57. doi: 10.1186/1750-1172-9-57 (PMC4021831; doi:10.1186/1750-1172-9-57)
Supplement: Additional file 2 — Targeted enrichment of known ataxia genes (“ataxia panel”). [file 1750-1172-9-57-S2.docx]

**Additional file 2: Targeted enrichment of known ataxia genes (“ataxia panel”)**

Sequencing started with fragmentation of DNA by digestion with multiple restriction enzymes. A set of gene-specific HaloPlex selector probes (Agilent, Santa Clara, CA, USA) was hybridized to the fragmented DNA, thereby inducing the target fragments to form circular DNA molecules that also contain method-specific sequencing motifs (PBS) as well as a biotin label. Probe/Target DNA molecules were captured and purified using magnetic streptavidin beads. Probe/Target circles wee then ligated to ensure that only perfectly hybridized fragments were circularized. A PCR amplification step provided an enriched library ready for sequencing. Sample barcodes (MID) were introduced during amplification for precise tracking. Finally, the enriched library was checked for quality and quantity prior to sequencing on an Illumina MiSeq sequencing device by means of a 2 X 150 bp paired end approach (MiSeq sequencing kit V2). Reads were mapped against the hg19 standard reference genome to detect SNPs, SNV, short deletions and insertions (SAMtools, IGV). Variants were annotated by comparing them to openly accessible data from the dbSNP and the 1000Genomes project (Annovar).
