# Supplementary material for: Phenotype and frequency of STUB1 mutations: next-generation screenings in Caucasian ataxia and spastic paraplegia cohorts
Source: Orphanet J Rare Dis. 2014 Apr 17;9:57. doi: 10.1186/1750-1172-9-57 (PMC4021831; doi:10.1186/1750-1172-9-57)
Supplement: Additional file 3 — Electropherograms of Sanger Sequencing. [file 1750-1172-9-57-S3.docx]

**Additional file 3: Electropherograms of Sanger Sequencing**

**
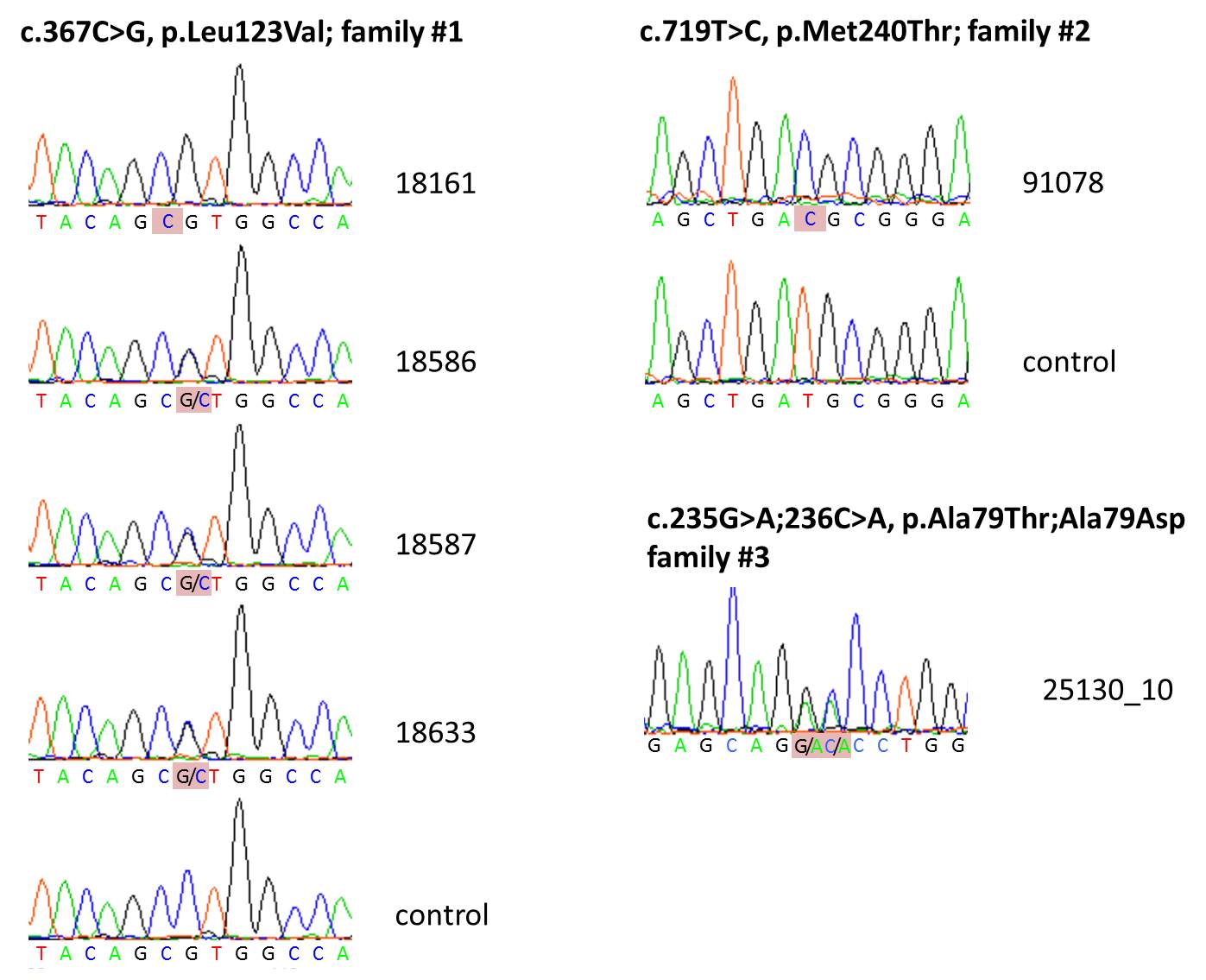
**

**Electropherograms of *STUB1* mutations identified with Sanger Sequencing.** The affected index patients 18161 (left column, top) and 91078 (right column, top) carry a homozygous nucleotid exchange, the affected index patient 25130_10 (right column, bottom) carries a compound heterozygous change.

Sanger sequencing of additional family members of subject 18161 (left column) (family #1) showed that the not-affected brother harbored the p.Leu123Val mutation in a heterozygous state, and testing of parents established both parents as heterozygous carriers.
