# Supplementary material for: Phenotype and frequency of STUB1 mutations: next-generation screenings in Caucasian ataxia and spastic paraplegia cohorts
Source: Orphanet J Rare Dis. 2014 Apr 17;9:57. doi: 10.1186/1750-1172-9-57 (PMC4021831; doi:10.1186/1750-1172-9-57)
Supplement: Additional file 4 — Sequencing Reads of an affected family member of family #3. [file 1750-1172-9-57-S4.docx]

**Additional file 4**

**
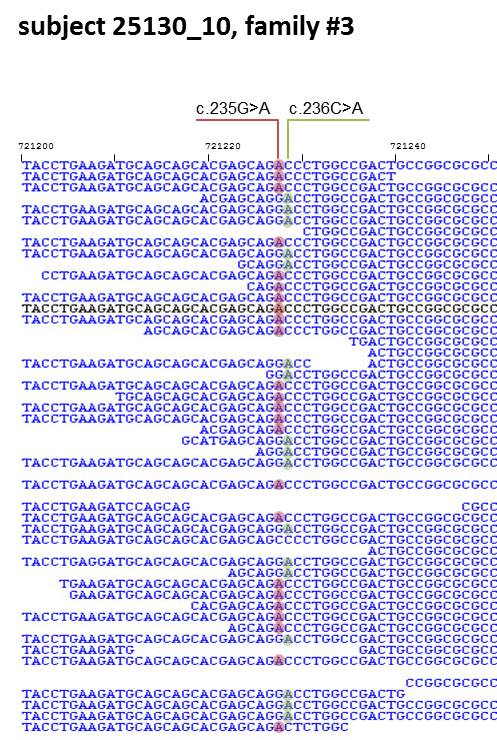
**

**Sequencing Reads of an affected family member of family #3.** Sequencing Reads of the index patient of family #3 (25130_10) were inspected manually using the BamView tool v1.2.10. The patient is heterozygous for two mutations in the *STUB1* gene that both affect codon 79 and are directly adjacent (chr16:731227G>A (c.235G>A, p.Ala79Thr) and chr16:731228C>A (c.236C>A, p.Ala79Asp). The screenshot depicts sequencing reads obtained for the genomic region chr16:731200-731250. Occurrence of the c.235G>A variant is marked by a red dot, occurrence of the c.236C>A variant by a green dot. Each of the sequencing reads carries only one of the mutations. This distribution strongly suggests that the two mutations are located on different alleles and therefore compound heterozygous.
