# Supplementary material for: Phenotype and frequency of STUB1 mutations: next-generation screenings in Caucasian ataxia and spastic paraplegia cohorts
Source: Orphanet J Rare Dis. 2014 Apr 17;9:57. doi: 10.1186/1750-1172-9-57 (PMC4021831; doi:10.1186/1750-1172-9-57)
Supplement: Additional file 5 — Genetic variants other than STUB1 identified in the STUB1 index patients by whole exome sequencing and the HaloPlex ataxia panel. [file 1750-1172-9-57-S5.docx]

**Additional file 5**

**Genetic variants other than STUB1 identified in the STUB1 index patients by whole exome sequencing (top) and the HaloPlex ataxia panel (bottom).** Subjects 91078 and 25130_10 were screened by whole exome sequencing, subject 18161 by a HaloPlex ataxia panel covering >120 known ataxia genes with a high coverage. Variants identified by WES were filtered according to the following filter criteria: minor allele frequency exome variant server ( MAF EVS6500) < 0.2%, number of segregating families within the in-house database GEM.app < 5; Genomic Evolutionary Rate Profiling (GERP) score > 2 or PhastCons > 0.4. Variants identified by HaloPlex ataxia panel were filtered according to the following filter criteria: minor allele frequency 1000 genomes < 1%, MAF EVS6500< 1%, coding change: not synonymous; number of samples within the in-house database <5; classification of the variant of unknown significance (VUS) >2. Explanation of further abbreviations: LRT, Likelihood Ratio Test; D, damaging; N, neutral. SIFT = Sorting Tolerant From Intolerant; SIFT scores <0.05 represent damaging effect.
