# Supplementary material for: Phenotype and frequency of STUB1 mutations: next-generation screenings in Caucasian ataxia and spastic paraplegia cohorts
Source: Orphanet J Rare Dis. 2014 Apr 17;9:57. doi: 10.1186/1750-1172-9-57 (PMC4021831; doi:10.1186/1750-1172-9-57)
Supplement: Additional file 6 — Heterozygous variants in STUB1 identified in a cohort of 1707 neurological disease controls (non-ataxia, non-HSP). [file 1750-1172-9-57-S6.docx]

**Additional file 6**

| **Genomic DNA change** | **CDS change** | **AA change** | **dbSNP137 ID** | **Phenotype** | **Segregation in family** | **MAF GEM.app** | **MAF EVS6500** | **PhastCons** | **GERP** | **PhyloP** | **Polyphen-2** | **LRT Class** | **Mutationtaster Class** | **SIFT score** |
| --- | --- | --- | --- | --- | --- | --- | --- | --- | --- | --- | --- | --- | --- | --- |
| chr16:g.731462G>T | c.383G>T | p.Arg128Leu |  | Autism | no | 0,058 |  | 0,968 | 4,59 | 2,366 | 0,932 | D | D | 0,04 |
| chr16:g.731813G>A | c.545G>A | p.Arg182Gln | rs145094142 | Deafness | no | 0,058 | 0,008 | 0,996 | 3,19 | 1,092 | 0,001 | D | N | 0,61 |
| chr16:g.731845G>A | c.577G>A | p.Val193Ile | rs200514887 | ALS (1 family) | n.a. |  | 0,015 | 0,004 | -4,48 | -0,721 | 0,007 | N | N | 0,44 |
| chr16:g.731855A>G | c.587A>G | p.Gln196Arg |  | ALS (1 family) | n.a. |  |  | 1 | 4,15 | 1,867 | 0 | D | N | 0,25 |
| chr16:g.732171G>A | c.676G>A | p.Asp226Asn |  | ALS (1 family) | n.a. |  | 0,015 | 0,888 | 5 | 2,469 | 0,617 | D | D | 0,04 |
| chr16:g.732392G>A | c.815G>A | p.Arg272Gln |  | ALS (1 family) | n.a. |  |  | 0,998 | 5,18 | 2,413 | 0,999 | D | D | 0 |

**Heterozygous variants in STUB1 identified in a cohort of 1707 neurological disease controls (non-ataxia, non-HSP).** The *STUB1* gene was screened for variants in 1707 whole exome sequencing datasets from families with neurological phenotypes other than ataxia and HSP. The following filter criteria were used: MAF EVS6500 < 3%, QUAL > 30, GQ > 30, < 10 additional families in the in-house database GEM.app with the same segregating variant. Positions refer to transcript NM_005861.2

**References for all additional files:**

1. Li H, Durbin R. Fast and accurate short read alignment with Burrows-Wheeler transform. Bioinformatics 2009;25:1754-1760.

2. DePristo MA, Banks E, Poplin R, et al. A framework for variation discovery and genotyping using next-generation DNA sequencing data. Nat Genet 2011;43:491-498.

3. McKenna A, Hanna M, Banks E, et al. The Genome Analysis Toolkit: a MapReduce framework for analyzing next-generation DNA sequencing data. Genome research 2010;20:1297-1303.

4. Gonzalez MA, Lebrigio RF, Van Booven D, et al. GEnomes Management Application (GEM.app): A New Software Tool for Large-Scale Collaborative Genome Analysis. Hum Mutat 2013;34:842-846.
